# General Practitioners’ Views on the Acceptability and Applicability of Using Quality Indicators as an Intervention to Reduce Unnecessary Prescription of Antibiotics in Four South American Countries

**DOI:** 10.3390/antibiotics7030057

**Published:** 2018-07-05

**Authors:** Inés Urbiztondo, Sandi Michele de Oliveira, Nieves Hernández-Flores, Lidia Caballero, Miguel Angel Suarez, Lars Bjerrum, Gloria Cordoba

**Affiliations:** 1 The Research Unit for General Practice and Section of General Practice, Department of Public Health, University of Copenhagen, 1014 Copenhagen, Denmark; inesurbiztondo@gmail.com (I.U.); lbjerrum@sund.ku.dk (L.B.); 2 The Institute for English, Germanic and Romance Studies, University of Copenhagen, 2300 Copenhagen, Denmark; smo@hum.ku.dk (S.M.d.O.); nhf@hum.ku.dk (N.H.-F.); 3 Dr. Pedro Baliña Hospital, Public Health Ministry, 3300 Posadas, Misiones, Argentina; lidia.gladis@gmail.com; 4 Policlínica Central de la Caja Nacional de Salud, La Paz 1086 Bolivia; sucumian@gmail.com

**Keywords:** antibiotics, quality indicators, general practice

## Abstract

As part of the quality improvement program “Health Alliance for Prudent Prescribing, Yield And Use of anti-microbial Drugs In the Treatment of respiratory tract infections” (HAPPY AUDIT) South America, we planned to implement an intervention based on the use of quality indicators as a means to influence General Practitioners’ (GPs) prescribing decision. Knowledge on the acceptability and applicability of an intervention is crucial to decide whether the intervention is suitable and will achieve the expected outcomes. This study explores GPs’ views about the acceptability and applicability of using quality indicators as an intervention to influence their prescribing decision in patients with suspected Respiratory Tract Infections (RTIs) across four countries in South-America. In March 2015, GPs that were participating in HAPPY AUDIT South America were invited to participate in focus groups. A discussion guide covering the domains acceptability and applicability was used. Data was analyzed through systematic text condensation with an inductive approach. 171 GPs were invited and 48% participated. Acceptability ranged from totally acceptable to slightly acceptable. This spectrum of GPs views on acceptability was influenced by themes concerning applicability. In conclusion, there is a positive attitude towards the use of quality indicators. Nonetheless, applicability challenges have to be taken into consideration and solved if we are to achieve a large effect with the implementation of this intervention.

## 1. Introduction

Antimicrobial resistance (AMR) is an increasing global problem, which represents a serious threat for societal development, due to its health and economic impact [1]. High consumption of antibiotics is associated with high prevalence of resistant strains [2]. Respiratory tract infections (RTIs) are the most common reasons for antibiotic prescribing in primary health care [3], even though most RTIs are caused by virus, and, in the majority of patients, antibiotics have no beneficial effect [4]. Decreasing the unnecessary prescription of antibiotics in patients with suspected RTI is crucial to curb the development of AMR.

Concrete actions need to be taken in South America. Not only is the prevalence of AMR increasing to alarming levels, but also the sale of antibiotics. For example, the prevalence of *S. pneumoniae* (the leading cause of community acquired pneumonia worldwide) resistant to penicillin in the American region has been estimated to be up to 68% [5], and the latest report about Global antibiotic consumption showed that South America is one of the main contributors to the global increase in antibiotic consumption [6].

Previous research has shown the importance of targeting interventions towards the general practitioners (GPs) to decrease the unnecessary prescription of antibiotics [7]. A qualitative study that was carried out in five European countries found that GPs prefer interventions that allow for comparison between colleagues [8]. Knowledge on local prescribing patterns triggers reflection and motivates change towards a more prudent use of antibiotics.

As part of the quality improvement program “Health Alliance for Prudent Prescribing, Yield and Use of anti-microbial Drugs In the Treatment of respiratory tract infections” (HAPPY AUDIT South America), we planned to implement an intervention based on the use of Quality Indicators (QI) as a means to influence GPs’ prescribing decision. QIs are expected to increase awareness of best practice, thus QIs have been advocated as a useful intervention to improve decision-making [9].

Previous research on interventions that are aimed at reducing the unnecessary prescription of antibiotics in primary care has shown that the success of any intervention depends largely on a thorough knowledge of the context [10], the acceptability of those exposed to the intervention, and the applicability of the intervention [11].

QIs as an intervention to decrease the unnecessary prescription of antibiotics have never been implemented in South America. Hence, we aimed to explore the acceptability and applicability of using QIs as a strategy to reduce the unnecessary prescription of antibiotics in patients with suspected RTIs seeking care in primary care within the South American context.

## 2. Results

### 2.1. Participating GPs

Of the 171 GPs invited to participate in the focus groups, 82 (48%) chose to do so. Table 1 shows baseline characteristics of the invited GPs divided by those who participated and those who did not. There were no statistically significant differences between participants and non-participants regarding distribution by gender or the proportion of high prescribers. GPs participating in the focus groups were younger than those who did not participate in the focus groups.

### 2.2. Acceptability Domain

Answers regarding the acceptability domain ranged from “totally acceptable” to “slightly acceptable”. The totally acceptable responses reflected a positive view of the QIs as an intervention that can guide them during the decision-making process as reminders of evidence-based practice:

“Yes… it would help us… it would work as a quick guide of good practice. During the consultation we [GPs] do not have a lot of time, so it would help to speed-up the decision-making process” FG_1_PGY

“It could help to modify our [GPs] prescription behaviour” FG_2_BOL

In the responses indicating moderate levels of acceptability, GPs kept a positive view, as well as a willingness to include consideration of quality indicators in their decision-making process. The GPs, however, also pointed out that the consultation process is complex, and there are clinical and non-clinical factors that could hamper a strict adherence to the indicators:

“Yes… We [the GPs] can use them [the QIs] … but in the end everything depends on the degree of certainty we [GPs] have about the diagnosis” FG_1_ARG

“We [GPs] could try to use them [QIs]… but we have to listen to our patients, too” FG_1_BOL

The “slightly acceptable” responses were those in which the GPs gave account of the contextual problems they face; none explicitly stated an aversion or unwillingness to integrate QIs in their decision to prescribe during the consultation.

“The indicators take into consideration the number of days [a patient has had] symptoms. If there is no certainty about the possibility for a follow-up in three or four days, I would prescribe antibiotics. I am sure that on Fridays, when Monday is a bank holiday, lot of antibiotics are prescribed” FG_1_PGY

“It will take time to get used to them [QIs] because we have never used clinical indicators… administrative… Yes…a lot… but clinical…” FG_1_BOL

### 2.3. Applicability Domain

Five main themes emerged regarding the applicability of the QIs, they are summarized in Table 2. The health system barriers theme was further divided into two sub-themes.

#### 2.3.1. Health System Barriers

● Accessibility

When GPs were asked about current prescription patterns and reasons for the lack of adherence to the proposed indicators, they mentioned that, often they would ask the patients to return for a second consultation a few days later. However, patients must have good access to the services that secure the close follow-up of care. In turn, if patients cannot access the healthcare centres, the GPs are hindered in their ability to adhere to the QIs; at that moment, they may be more likely to prescribe antibiotics rather than taking a more conservative approach.

“I am personally against the liberal prescription of antibiotics. However, when I have a patient with a suspected acute otitis media I prescribe antibiotics straightway regardless age or number of days with symptoms. Lots of my patients live at least 12 kilometers walking [distance] from the health care centre. If the symptoms get worse, they cannot come back” FG_1_PGY

“The indicators can be used… provided the patient has good accessibility to health care services” FG_3_ARG

● Over-the-counter antibiotics

Over-the-counter sale of antibiotics is common within the South American context. The possibility to get antibiotics without a prescription decreases the societal impact of the efforts made by the GPs by bearing in mind the indicators to guide their prescribing decision.

“The QIs can be used… but there is a revolving door… then… the final effect on antibiotic consumption at country level is not too high” FG_2_BOL

“The pharmacist will never say NO to the opportunity of selling an antibiotic” FG_2_ARG

“Most children do not need antibiotics. The problem is the unmet expectations from the mother. The mother gets angry because her child did not get antibiotics…. And at the end, the mother just goes and buys them [antibiotics] at the pharmacy… wrong dosage… a mess” FG_2_BOL

#### 2.3.2. GPs’ Considerations about Belonging to a Professional Group

Study participants expressed the need for consensus among colleagues as a facilitator for the applicability and long-term use of the QIs during the consultation. The QIs should be endorsed by the professional groups, backing-up the homogenization of clinical criteria.

“It is easier to be part of the 95% of doctors that freely prescribe antibiotics, than being part of the 5% of doctors that have a more restrictive use of antibiotics” FG_1_ARG

“I do not think that the use of indicators is negative as a concept…as an idea…, but it will be very difficult to succeed in the widespread use of the indicators. It requires that we [GPs] unify clinical criteria... and it is difficult” FG_2_BOL

#### 2.3.3. Lack of Diagnostic Tools

The limitations in availability of diagnostic tools in many healthcare centres cause uncertainty in the diagnosis and affect the decision of whether to give antibiotics or let the disease follow its natural course.

“The problem is that we do not have access to diagnostic tools that help us to convince/negotiate with the patients” FG_1_PGY

“The diagnostic tools needed to differentiate between pneumonia and bronchitis are not available” FG_1_ARG

“Lot of health care centres are far away from urban areas. In those centres, the only diagnostic tools we (GPs) have are ourselves and a stethoscope… nothing else” FG_2_UGY

#### 2.3.4. The Doctor-Patient Relationship

The applicability of the indicators depends on a good and trustworthy doctor-patient relationship. This relationship is perceived as a means to guarantee that the message is understood and implemented by the patient. The patient believes and understands what the GP wants to communicate whether both participants have taken the time to get to know each other. No less important is the doctor’s knowledge of the patient’s ability and willingness to follow the treatment as prescribed.

“The indicator works, provided one has a good patient-doctor relationship because one can be sure that the patient understands the message” FG_3_ARG

“I think too that the use of indicators depends a lot on our knowledge about the patient. It will mean taking more time during the consultation to convince the mother that her child does not need antibiotics and that there are options such as waiting 3 days to assess evolution. As Dr X said, if one knows one’s patients, if one knows what they think, how to talk to them… one can convince them [the patients]” FG_1_BOL

#### 2.3.5. Content/Face Validity of the Indicators

The indicators cannot be implemented without prior tailoring to the GPs population. GPs recognized and pointed out heterogeneity across and within countries and emphasized the need to consider the socio-economic factors from the patients when assessing the quality of the prescription of antibiotics.

“We [the GPs] know our patients, … they [the patients] are different from the European population. There is malnutrition, illiteracy, a very weak immunological system. The evidence from European studies cannot be applied to our population” FG_2_BOL

“I think the indicators need to discriminate between rural and urban practice due to problems of access [to healthcare]” FG_2_PGY

## 3. Discussion

### 3.1. Main Findings

In this study, we found that GPs have a favorable outlook towards the use of quality indicators as an intervention to reduce the unnecessary prescription of antibiotics in patients with suspected RTI seeking care in primary care. Nevertheless, the application of such practices, and thus their acceptance by GPs, is generally affected by challenges in the applicability of the quality indicators based on a variety of contextual factors.

### 3.2. Strengths and Limitations

The major strength of the study is its focus-group approach. The group context made it possible to capture the range of views on acceptability and applicability challenges [12], and the face-to-face discussions between the participants triggered a wide spectrum of answers about the acceptability and the applicability of the QIs as an intervention to influence the prescribing decision.

Another strength of the methodology derives from the fact that three out of the four interviewers were not working within the primary health care level in any of the participating countries. As a result, the GPs took an open and broad view about the topics arising during the session, and there was no risk of bias due to interviewers driving the discussion towards their own experiences [13].

The use of a semi-structured interview guide allowed for consistency in the development of the focus groups. Similar phrasing of the guiding questions and discussion of the dynamics and answers of the focus groups guarantee that the responses between the groups can be compared [14].

The main limitation of this study is the extent of transferability of our findings. Previous studies have demonstrated that GPs participating in audits and research have a lower tendency to prescribe antibiotics [15,16]. Hence, those who agreed to participate may have a particular interest in judicious antibiotic prescribing or be less likely to prescribe antibiotics. Still, given that a quarter of the participants in this study were classified as high-prescribers, the bias towards less frequent prescribers may not be great. Furthermore, those participating in the focus groups tended to be younger than those who did not accept the invitation. Previous research has shown that younger GPs adhere more frequently to guidelines and tend to prescribe less antibiotics [17]. It could explain the positive views towards the use of quality indicators. Nonetheless, the wide range of responses shows that age may not have played a role in the final results. Due to the focus group dynamic, it is not possible to know whether there is a difference in the range of views regarding acceptability, depending on the age of the participant.

Secondly, participants may have been constrained by the content of the interview guide or given socially desirable answers. The first three groups were divided into high and low prescribers as well as by age. All of the participants had the opportunity to express their opinions, and we did not find difference in the range of answers.

### 3.3. Comparison with Previous Research

To our knowledge, this is the first study exploring GPs’ views on the acceptability and applicability to use QIs as an intervention to reduce the unnecessary prescription of antibiotics within the South American context. In this case QIs were the proposed intervention, but the answers regarding acceptability and applicability could be transferable to other types of interventions that are aimed at reducing the unnecessary prescription of antibiotics in patients with suspected RTI in primary care.

For example, participating GPs were concerned about the accessibility of patients to healthcare facilities. Consequently, GPs would rather prefer to prescribe antibiotics than bearing in mind the QIs. Our findings are in agreement with previous research in which GPs prefer to take a “no risk” action if they are not sure the patient has the possibility to come back for a closer follow-up [18,19,20].

In line with studies from other low middle-income countries (LMICs) [11,21], over-the-counter sales of antibiotics were mentioned as an important problem. GPs that were participating in our study said that, even if they decide not to prescribe antibiotics, the patient could go to the pharmacy and buy the antibiotic without prescription. This was the case in the four countries, even though three of the four (Bolivia being the exception) have clear regulations banning the availability of antibiotics without a prescription. As GPs from all countries mentioned that over-the-counter antibiotics continue to be a common problem in everyday practice, our study points to the need to further examine and strengthen the mechanisms that are designed to restrict patient access to antibiotics without a prescription.

The doctor-patient relationship is another central point that has been mentioned by GPs in another settings [22,23,24]. GPs prioritize empathy towards the patient above the societal problem of antimicrobial resistance. A close doctor-patient relationship is considered to be crucial to secure good communication in which the message is not only well understood but is accepted by the patient. A recent systematic review has shown that interventions that are aimed at facilitating shared decision making (between doctors and patients) reduce antibiotic prescribing in primary care [25]. For example, a trial assessing the effectiveness of enhanced communication skills and use of C-reactive protein against “usual” care found that enhanced communication reduced the proportion of antibiotic prescribing when compared with usual care. Enhanced communication coupled with use of C-reactive protein resulted in the highest decrease in prescriptions [26].

As the point of entrance to the health care system, GPs see patients with very unspecific symptoms. Similar to our findings, several studies have found that GPs prescribe antibiotics due to the uncertainty about the correct diagnosis. In many cases, GPs do not have access to diagnostic tools, and cannot rule out the bacterial origin of the symptoms [18,22,27,28]. QIs rely on a correct labelling of the diseases; consequently, without the right diagnostic tools, it is difficult to adhere to the QIs.

Finally, GPs work in “communities of practice”, combining information from a wide range of sources into “mindlines” (internalized, collectively reinforced tacit guidelines), which they use to inform their practice [29]. This has important implications for the applicability of the QIs. GPs in our study commented that the lack of consensus among doctors makes their individual decision not to prescribe antibiotics harder, because even if they decide not to prescribe antibiotics, other doctors will. This also sends mixed signals to the patients, who may seek out willing doctors if their own denies them a prescription. QIs cannot be implemented if there is not a professional consensus and acceptance that all GPs should adhere and use them.

### 3.4. Relevance and Further Research

During the focus groups, the GPs claimed that QIs that were developed in Europe cannot be applied within the South American context; a qualitative study from India found similar results [18]. In both contexts, GPs cite unhygienic conditions, poor immune system and a higher risk for bacterial infections as parameters that need to be taken into consideration in the development and implementation of the QIs. Most of the evidence, if not all, comes from high-income countries with lower prevalence of antibiotic resistance and different health seeking behavior (i.e., patients consulting with more than two days with symptoms).

Differences in sanitary conditions and higher prevalence of resistant strains might imply that the current evidence about the high number that is needed to treat to benefit one is not fully applicable in LMICs. Political commitment to strengthening antibiotic stewardship and research development at the primary health care level is crucial in order to succeed in the final goal of decreasing the unnecessary prescription of antibiotics in LMICs, then contributing to curb the development and the spread of antimicrobial resistance.

Recent research in Spain found that each of the factors illustrated in this South American study is also operative in Spain [30]. This suggests that continued comparative qualitative research can be of value, particularly when the cultural heritage context is similar.

Furthermore, antimicrobial prescribing in primary health care level occurs within the context of a wide social network with multiple actors who continuously interact [31,32,33,34]. Lack of engagement with this broader group (e.g., GPs, pharmacist, nurses) may fail to account for what truly influences prescribing practices, and, more importantly, fails to deliver interventions that optimize prescribing behaviors [35].

## 4. Materials and Methods

### 4.1. Study Design

An explorative qualitative design using focus groups was carried out to investigate the acceptability towards using quality indicators as an intervention to reduce the unnecessary prescription of antibiotics in patients with suspected RTI seeking care in primary health care. Focus groups were preferred over single interviews to encourage discussion among participants about their different points of view.

### 4.2. Setting and Participants

General practitioners from Argentina, Bolivia, Paraguay, and Uruguay participating in the quality improvement program HAPPY AUDIT-II (Health Alliance for Prudent Prescribing, Yield And Use of anti-microbial Drugs In the Treatment of respiratory tract infections) were invited to participate in the focus groups [36].

In March 2015, the GPs were invited to a four-hour meeting in each country. In the first part of the meeting, they received quantitative feed-back on their prescription practices. The second part of the meeting was reserved for the focus group interviews. In total, there were nine groups, with a maximum of ten GPs per group.

In order to stimulate discussion, the first three groups were organized by the age and prescription pattern (high or low in relation to their media), but after comparing the topics emerging from these groups, it was decided to organize the groups randomly.

### 4.3. Data Collection—Focus Group Development

A discussion guide was developed by GC-LC-LB. The discussion guide followed a funnel design, whereby the discussion flowed from broad questions to specific ones. The guide covered two domains: (a) acceptability and (b) applicability. The specific questions took as a reference point QIs developed in 2010 for the EU-funded project HAPPY AUDIT [37] and QIs that were developed by European Surveillance of antimicrobial consumption (ESAC) [38] (see questionnaire Supplementary File 1). The quality indicators discussed during the focus group were:

- Percentage of patients aged two or older, with symptoms present for fewer than three days and a diagnosis of acute otitis media, who were prescribed antibiotics—standard 0–20%
- Percentage of patients with symptoms lasting fewer than five days and diagnosed with rhino sinusitis, who were prescribed antibiotics—standard 0–20%
- Percentage of patients diagnosed with acute bronchitis, who were prescribed antibiotics—standard 0–30%
- Percentage of patients with a suspected viral respiratory tract infection, who were prescribed antibiotics—standard 0–5%

We sought to enhance the rigor of the data collection by: (a) following the standardized discussion guide, (b) digitally recording the focus groups, and (c) triangulating information at the end of each focus group discussion, as the moderators met and discussed the main topics that were covered and the interactions of the participants.

### 4.4. Analysis

The focus group discussions were recorded digitally and transcribed fully. The corpus for analysis encompassed two hours per focus group for a total of 18 h of data. To ensure that the content was in line with the discussion, the transcriptions were read by the moderators of the focus groups.

Qualitative content analysis was carried out using an inductive approach [39] based on Giorgi’s psychological phenomenological analysis [40]. That means that, for the first phase of the data analysis to get an overview of the data, we tried to remain as atheoretical as possible to secure a rigorous shift between the decontextualization and contextualization of the data. GC, NHF, and SMO have experience in doing research in prescription of antibiotics in primary care. To secure reflexivity, they read the first four transcripts independently and compared their findings to objectively assess whether preconceptions or specific theory would be guiding the process. Afterwards, the transcripts were coded line by line. These codes were compared for similarities and differences in order to come up with the first list of themes. Each researcher listed the emerging themes and through discussion agreed on preliminary themes. GC coded the remaining focus groups, and together with NHF and SMO, agreed on the second list of themes.

GC identified quotes in all of the transcripts reflecting each theme and developed preliminary sub-themes. From the corpus, selected statements were extracted for their representativity of the defined categories. The final decisions on themes, sub-themes, and descriptions were conducted through discussions and agreements.

Four of the authors of this article are GPs who participated in the study; each read and approved the interpretation of the content of the focus groups. Afterwards, GC and SMO translated the quotations and themes into English, and added clarifying information in square brackets (e.g., “they [the patients]”.

### 4.5. Ethics

Ethical approval was granted in each country by the following authorities: Bioethics committee, Posadas, Misiones (Argentina) (File Nº 022014). Department of Quality, Education and Research at “Caja Nacional de Salud” La Paz (Bolivia) (File Nº 29/05/2014) and ethics committee from “Arco Iris” Hospital. Ministry of Health and welfare, Seventh Health Zone, Encarnación (Paraguay) (File Nº 116/2014). Ethical committee for research projects at the Faculty of Medicine, University of the Republic, Montevideo (Uruguay) (File Nº 070153-000309-14).

## 5. Conclusions

This study identifies a positive attitude towards the use of QIs among GPs that are working within the South American context. However, there are a number of factors that need to be in place to include QIs as effective tools for improving and harmonizing GP prescription practices: (a) Health system barriers; (b) the GPs’ evaluation of the importance of belonging to a professional group; (c) the appropriate procedures for determining appropriate decision-making processes; (d) the establishment and maintenance of a good doctor-patient relationship; and, (e) the inclusion of content and face validity considerations of the indicators. Therefore, other initiatives should be evaluated before implementing QIs as a tool to decrease antibiotic prescribing in South America. These include adopting stronger regulations and ensuring the reliable enforcement of the laws prohibiting the over-the-counter sale of antibiotics, delayed prescribing of antibiotics in cases where accessibility is an issue (the doctor makes a prescription, but recommends the patient to wait before starting the antibiotic treatment to see if the symptoms are self-limited), or patient education in the pharmacies when the patients ask for over-the-counter antibiotics, among others. Future research should focus on assessing the feasibility and effectiveness of the aforementioned initiatives.

## Figures and Tables

**Table 1:** Baseline characteristics of the invited General Practitioners (GPs0).

	Participants N = 82	Non-Participants N = 89	Invited N = 171
**Female (%)**	53 (64)	64 (71)	117 (68)
Age (SD) *	36 (7.2)	41 (9.8)	38 (8.8)
**High prescribers ^¥^**	22 (26.8)	27 (30.3)	49 (28.6)
Years as practitioner *	7.2(6.2)	9.9 (8)	8.5 (7.4)

* Mean (SD) = Standard deviation. ^¥^ High prescribers = ^¥^ GPs prescribing antibiotics to more than 75% of their patients diagnosed with RTIs.

**Table 2:** Emerging themes and subthemes regarding applicability.

**Health System Barriers**
● Accessibility
● Over-the-counter antibiotics
**GPs as a professional group**
**Decision-making process**
**Doctor-patient relationship**
**Content and Face validity of the QIs**

## Supplementary Materials

The following are available online at http://www.mdpi.com/2079-6382/7/3/57/s1.

## Abbreviations

FG	Focus Group
the number	the number of the focus group
PGY	Paraguay
BOL	Bolivia
ARG	Argentina
UGY	Uruguay
