# Supplementary material for: General Practitioners’ Views on the Acceptability and Applicability of Using Quality Indicators as an Intervention to Reduce Unnecessary Prescription of Antibiotics in Four South American Countries
Source: Antibiotics (Basel). 2018 Jul 5;7(3):57. doi: 10.3390/antibiotics7030057 (PMC6163160; doi:10.3390/antibiotics7030057)
Supplement: Supplementary file 1 [file antibiotics-07-00057-s001.pdf]

Guide

1. Do you think that using quality indicators would help you make a better use of antibiotics?

Acute Otitis Media

2. What are the reasons for prescribing antibiotics to a patient older than 2 years with less than 3 days with symptoms in whom you suspect an otitis media?
3. Now XX% of the patients older than 2 years with less than 3 days with symptoms and with otitis media are prescribed antibiotics. Do you think that a standard of 20% maximum could be implemented in your practice?

Rhinosinusitis

4. What are the reasons for prescribing antibiotics to a patient with less than 5 days with symptoms with a presumable diagnose of sinusitis?
5. Now XX% of the patients with less than 5 days with symptoms with a presumable diagnose of sinusitis are prescribed antibiotics. Do you think that a standard of 20% maximum could be implemented in your practice?

Acute bronchitis

6. What are the reasons for prescribing antibiotics to a patient with a diagnose of acute bronchitis?
7. Now XX% of the patients diagnosed with acute bronchitis are prescribed antibiotics. Do you think that a standard of 30% maximum could be implemented in your practice?

Viral origin symptoms

8. What are the reasons for prescribing antibiotics to a patient whose symptoms, you judge, are likely of viral origin?
9. Now XX% of the patients with symptoms with a presumable viral origin are prescribed antibiotics. Do you think it is possible to reduce that precentage?

10. Do you have any further considerations about why these 4 indicators can or cannot be used for the evaluation of appropriate antibiotic use in patients with respiratory symptoms in your everyday practice?
